# Development of an Arabic inpatient satisfaction survey: application in acute medical rehabilitation setting in Saudi Arabia

**DOI:** 10.1186/s12913-017-2596-2

**Published:** 2017-09-18

**Authors:** Ahmed Aboabat, Hazem Qannam

**Affiliations:** 1 Rehabilitation Hospital, King Fahad Medical City, P.O. Box 69762, Riyadh, 11557 Kingdom of Saudi Arabia; 2 Rehabilitation Hospital, King Fahad Medical City, P.O. Box 59046, Riyadh, 11525 Kingdom of Saudi Arabia

**Keywords:** Patient satisfaction, Rehabilitation, Survey development, Psychometric properties

## Abstract

**Background:**

In the management of chronic disease, evidence suggests that satisfied patients exhibit more loyalty to treatment providers and greater adherence to treatment regimens. This is particularly so in the rehabilitation setting. We aimed to develop a reliable and valid Arabic-language survey to objectively measure inpatient satisfaction in medical rehabilitation settings in Saudi Arabia.

**Methods:**

The King Fahad Medical City Rehabilitation Hospital Patient Satisfaction Survey (RH PSS) is a self-administered survey that addresses four domains of rehabilitation care: access, structure, process, and outcomes. The RH PSS was developed through four steps. Step 1: An item-generation process utilizing input from patients, rehabilitation professionals, and the relevant literature. Step 2: Individual interviews and focus groups, conducted for cognitive testing of the survey and to examine content validity. Step 3: Assessment of internal consistency and construct validity. Step 4: Survey implementation wherein factor analysis and reliability and validity testing were conducted. The survey was conducted at an acute inpatient medical rehabilitation hospital in Saudi Arabia. A total of 709 rehabilitation inpatients participated.

**Results:**

The RH PSS demonstrated reasonable reliability and validity. Cronbach’s alpha for all the RH PSS subscales ranged from 0.81 to 0.89, and 0.96 for the entire survey. Factor analysis showed good correlation of the 33 survey items and the subscales. The RH PSS demonstrated a good level of predictive validity through the high correlation between the global item “intent to recommend” and overall satisfaction (R2 = 0.786, adjusted R2 = 0.783, *p* = 0.01).

**Conclusions:**

The RH PSS is the first satisfaction survey with reported validity and reliability testing to address inpatient rehabilitation settings in Saudi Arabia. Further research involving multiple sites is recommended for nationwide validation.

**Electronic supplementary material:**

The online version of this article (10.1186/s12913-017-2596-2) contains supplementary material, which is available to authorized users.

## Background

The patient satisfaction survey is among the most commonly used outcome measures in the health care industry. The results of these surveys are increasingly considered a primary health care outcome, with results considered an important quality indicator in program reviews, accreditations, and funding [1–6]. Available evidence suggests that satisfied patients are more likely to be loyal to treatment providers and adhere to treatment regimens. Conversely, dissatisfied patients may harbor and share negative impressions with friends, physicians, or funding agencies and this may become an obstacle to full utilization of services [7].

Patient satisfaction is particularly vital to the success of rehabilitation for a number of reasons: (i) the active participation of the patient is critical for improvement and learning of new skills; (ii) the full attention of the patient and his/her family are critical for the effective management of chronic disease conditions; and (iii) sustained cooperation of the patient is essential because of the longer duration of rehabilitation compared with other medical settings [3]. Indices of patient satisfaction in Saudi Arabia will inform clinicians and program managers in their endeavors to improve services and assist marketing personnel with the information needed to attract clients [8].

Patient satisfaction information is increasingly used in funding decisions by health authorities. In a study by Campbell et al. [5], the United Kingdom National GP Patient Survey reported that survey findings led to the distribution of £65 million of UK National Health Service (NHS) resources in 2008/9. In the US, 1% of Medicare payments have been linked to hospital quality performance, with one third of this amount based on how well hospitals score for patient satisfaction [9].

Professional organizations and accreditation bodies increasingly recognize the importance of patient satisfaction as a key element in the success of rehabilitation programs. One such organization, the Commission on Accreditation of Rehabilitation Facilities (CARF), requires that rehabilitation facilities record patient perception of the rehabilitation experience. This provides a vital outcome measure and so constitutes a core component of the standards of excellence in the service provided [6].

The complex, multidimensional nature of patient satisfaction has led to the development and use of several measurement approaches (suggestion boxes, formal complaints, qualitative methods, and audits), yet questionnaires remain the most commonly used [10] because they are convenient to prepare. However, the subjective nature of patient satisfaction assessment continues to attract debate as to its benefit and usefulness [11]. This debate often concerns the appropriate use of specific variables, highlighting that questionnaires must have specific psychometric properties to effectively generalize the information to the target population [11, 12].

While patient satisfaction surveys can involve one or two questions about overall or general satisfaction, or can be multidimensional, containing various items probing different aspects of satisfaction, most address a relatively common set of issues. Indeed, after analyzing the content of 111 theoretical and empirical articles related to patient satisfaction, Ware et al. noted eight common domains: art of care, technical quality, accessibility/convenience, financial aspects, physical environment, availability of providers and facilities, continuity of care and efficacy/outcomes, with the first four being the most common measures for healthcare [13].

Literature in the field of rehabilitation reports differing results, correlating satisfaction with various demographic factors, structures, processes, and therapy outcomes. Satisfaction with rehabilitation may be influenced in part by age, with older adults being more satisfied, but other demographic characteristics not affecting satisfaction [4, 14]. However, some authors [15–17] have reported that demographic variables including age were not predictors of levels of satisfaction. In a Canadian rehabilitation setting [18], a study conducted in 2001 found that clients were satisfied with the accessibility, quality, and outcomes attributed to occupational therapy. A 2006 analysis of data from patient surveys in German rehabilitation hospitals found that satisfaction with the general atmosphere of the hospital, the medical care, and the success of rehabilitation at discharge were the strongest factors contributing to satisfaction, particularly concerning a patient’s willingness to recommend a hospital [19]. In other research, satisfaction was found to be inversely correlated with pain and active range of motion in patients with chronic neck pain [20], hip or knee replacement surgery [17] with increased pain resulting less satisfaction [19].

A systematic review of studies conducted in English-speaking countries, reported that the interpersonal attributes of therapists and the process of care were key determinants of patient satisfaction, while the actual treatment outcome was surprisingly not a consistent determinant of satisfaction with physical therapy care [21]. However, Mancuso et al., found that Functional Independence Measure (FIM) motor scores suggesting greater mobility at discharge in patients with multiple orthopedic impairments were the strongest predictor of satisfaction in the total sample [4]. Similarly, patients with cerebrovascular impairment who had a higher FIM total rating at discharge were found to be more satisfied [22].

Despite the wide use patient satisfaction surveys and an increase in the number of studies in the field of patient satisfaction, the application of surveys, the number of published studies, and the consistency of the reported findings in the field of rehabilitation continue to be somewhat limited [3, 9, 14]. There is no gold standard for assessing patient satisfaction [23], and the field of rehabilitation continues to require standard and validated measurement tools to assess patient satisfaction in various settings [3, 14]. Moreover, currently-available surveys, having been developed mostly in North America and Europe, pose challenges for effective use in Saudi Arabia because of differences in language, culture, and health systems.

The aim of this study was to develop a valid and reliable Arabic survey that could meaningfully measure the satisfaction of inpatients in medical rehabilitation settings in Saudi Arabia. We describe herein the development of and validity/reliability assessment of the King Fahad Medical City-Rehabilitation Hospital Patient Satisfaction Survey (RH PSS).

## Methods

There were four steps in the development and implementation of the survey. The process of survey development occurred in 2010 and incorporated the first three steps: (1) undertaking semi-structured interviews with rehabilitation inpatients and healthcare professionals, and consulting the relevant literature, (2) conducting focus groups with inpatients and rehabilitation professionals to evaluate the perceived importance of the specific survey items and associated domains for inclusion in the survey, and (3) exploring the instrument reliability and validity and performing factor analysis. The fourth step involved implementation of the survey.

The protocol was approved by the Institutional Review Board of King Fahad Medical City. The survey was distributed to all KFMC Rehabilitation Hospital (KFMC-RH) inpatients upon discharge. All inpatients provided informed consent, or in the case of those younger than 18 years of age, assent, prior to participation.

### Step 1: Development of a preliminary instrument

To determine the contents and items that cover the construct of interest thoroughly, we performed semi-structured interviews with inpatients at KFMC-RH lasting up to 30 min. Interviews were constructed using open-ended questions to encourage free discussion. Participants were asked to define their level of satisfaction, elaborate on their current experience and expectations, and provide expressions that could be used to develop questions for the survey. Interviewers continuously probed participants for additional information. Content analysis of the interview transcripts was completed, and the identified themes provided an initial list of specific survey items. Next, a literature search was conducted to analyze available patient satisfaction surveys to identify domains and specific satisfaction survey items that did not emerge from the interviews [24–28]. Finally, content analysis and consensus-oriented review was conducted through a focus group with rehabilitation professionals who were asked and prompted to provide opinions related to what they perceived important for inclusion in a patient satisfaction survey.

### Step 2: Cognitive testing of the survey

The first draft of the survey was piloted with five patients who were then interviewed to probe their understanding of each survey item and the chosen response. Participant responses were noted, and modifications were made accordingly.

Two rounds of focus groups were convened with rehabilitation professionals of diverse specialties to establish the face validity of the first draft of the survey [29, 30]. A CARF expert reviewer also participated in this step. In the first round, we facilitated a semi-structured group discussion during which participants were asked to rate item importance, their interpretation and understanding of each item, the rating scale, and the format and wording. They were also asked to suggest new items or comment on the need to introduce new items. During the second round, we facilitated a semi-structured group discussion of each domain and item in the survey. Participants were probed on wording, omissions, or the addition of new items. Modification to each domain and survey item was made accordingly, until consensus was reached.

### Step 3: Preliminary validation & survey finalization (2010)

The survey items were subjected to Classical Test Theory (CTT) procedures using the Statistical Package for the Social Sciences (SPSS, version 18). Descriptive statistics were completed to characterize participant demographics and used to examine the correlation between participants’ demographic characteristics and the satisfaction rate. We assessed the internal consistency and reliability of the survey using Cronbach’s alpha [31]. Alpha levels were interpreted as: unacceptable (<0.60), undesirable (0.60–0.65), minimally acceptable (0.65–0.70), respectable (0.70–0.80), and very good (0.80–0.90) [32]. To measure concurrent validity, any new survey and the gold standard are administered at the same time [30]. In the absence of a gold standard, we assessed predictive validity similar to other researchers [6, 21], through establishing correlation between all the survey items and the global item “I would recommend this program to a friend or family member.” Pearson correlation and regression analysis were performed to establish such correlations.

Factor analysis was used to test the survey construct validity and to determine the number of factors with corresponding items on the final survey [32]. First, the Kaiser–Meyer–Olkin (KMO) measure and Bartlett’s test of sphericity were used to confirm that there was good inter-correlation between items (KMO statistic >0.60) and whether the population correlation matrix was identical (Bartlett’s test was significant at *p* < 0.05). Principal components exploratory factor analysis with Varimax rotation was then performed to determine the number of factors with corresponding items in the list of specific goals with an eigenvalue >1. Items that loaded onto a factor with a variance >0.30 were grouped together. If there was multiple loading of an item onto several factors, the item was grouped with the factor with a greater conceptual relationship. Groupings were checked for content validity.

### Step 4: Survey implementation

Upon survey implementation with a larger sample, over 4 years, the survey items were again subjected to CTT procedures, e.g., Cronbach’s alpha (internal consistency measure for instrument reliability), factor analysis for construct validity, and multiple regression analysis for predictive validity. To examine the relationship between the study participants’ demographics and the satisfaction rate, one way ANOVA with Tukey test and Pearson correlation were used. This analysis involved 709 surveys completed between 2010 and 2013.

## Results

### Step 1: Development of a preliminary instrument

The content analysis of the interviews with patients, literature review and the consensus-oriented focus group with rehabilitation professionals yielded an initial list of 49 items listing different aspects of the inpatient medical rehabilitation that may have a bearing on patient satisfaction (Table 1). All items were worded as statements.

**Table 1:** Items development and modification according to various steps of the survey development

Survey development			Modification		
Theme	#	Survey item	Step 1^a^	Step 2^a^	Step 3^a^
Pre-Admission Phase	1.	Waiting time to be admitted	N	M	
		Time you had to wait for a bed (after arrival at the hospital)	N	R	
	2.	Info re: rights & responsibilities	N		
	3.	Clarity of info about your admission	N		
		Courtesy and helpfulness of the admission office staff	N	R	
		Way team involved you in the decision to be admitted	N	R	
Admission Phase	4.	Way staff involved you in making decisions about your program	N	M	
	5.	Way team considered your needs	N	M	
		Being encouraged by the team to give your feedback	N		R
	6.	Clarity of info about your rehab program	N		
	7.	Knowing who to ask when you have questions	N	M	
		How well the team responded to your questions	N		R
	8.	Courtesy and helpfulness of your team	N	M	
		Courtesy and helpfulness of your (doctor, nurse, PT, OT, SLP, P&O, psych, support workers …etc. ) – one question each	M	R	
		Cooperation and commitment of your rehabilitation team	M	R	
	9.	Efforts made by your rehab team	M	M	
		Efforts made by your (doctor, nurse, PT, OT, SLP, P&O, psych, support workers …etc. ) – one question each	N	R	
	10.	How soon did nursing respond to your call for help	M	M	
		Responsiveness of your (doctor, nurse, PT, OT, SLP, P&O, psych, support workers …etc. ) to your needs –one question each	N	R	
		Confidence and trust in your doctors, nurses, therapists	N	R	
	11.	Communication between the team	N	M	
	12.	Explanation about your medications	N		
		The team response to your complaints (if any)	N	R	
	13.	The daily rehabilitation routine	N	M	
	14.	Length of your rehab program	A	N	
Physical Environment		Cleanliness of your room	N	M	R
	15.	Privacy in your room	N		
	16.	Cleanliness of hospital	N		
	17.	Cleanliness of the toilets and showers	N		
	18.	Quality of food overall	N		
	19.	Peace and restfulness in your room	N		
	20.	That your safety was not compromised	N	M	
Predischarge Phase	21.	Meeting to discuss your discharge plans	N		
		Info received about home medication	N	M	R
		Info re: home exercise program/ how to care for yourself at home	N		R
Discharge Phase	22.	Way & time given to planning your return to home	N		
	23.	Arrangement by the hospital for services/technical aids	N	M	
	24.	Arrangement for needed follow up plans	N	M	
		Info about how to manage your condition and recovery at home	N	M	R
		Info re: activities you could/ could not do on your own at home	N	R	
Outcomes		Level of satisfaction with improvement in body function e.g. range of motion, muscle power, tone, upper/lower extremity function…etc.	N	R	
		Level of satisfaction with functional mobility e.g. walking, wheelchair, indoor, outdoor, up/down stairs …etc.	N	R	
		Level of satisfaction with your ability to perform self-care tasks (e.g. eating, grooming dressing, …etc.); school, work; leisure…etc.	N	R	
		How much you were actually helped by your stay in the hospital	N	R	
		Know where and how to find help in the community		A	R
	25.	My physical pain was controlled	N	M	
	26.	I was given adequate info about medicines		A	
	27.	Given adequate info re: changes to my home	M		
	28.	I / My family/caregiver received adequate information/ training in order to manage my condition and recover at home.		A	
	29.	I accomplished the goals set in my rehab		A	
	30.	Confident in my ability to use the skills I was trained in		A	
Global Questions		Level of satisfaction with the care you received from (doctor, nurse, PT, OT, SLP, P&O, psych…etc.) – one question each	N	R	
	31.	I think this hospital has everything needed		A	
		Return to this facility if you require future rehab	M	R	
	32.	I would recommend this program	N	M	
	33.	Overall, I was satisfied with my experience	N		
Open-ended questions	What could the hospital do to improve the care and services it provides to better meet the needs of the patients		N	M	

^a^Changes to the items during these steps: *A* added, *N* no change, *R-* removed, *M* modified wording; items highlighted in bold text represents those items included in the final survey

### Step 2: Cognitive testing of the survey

Eleven rehabilitation practitioners participated in the first round of the focus group. These were one physiatrist, one nurse, three physical therapists, two occupational therapists, one speech and language pathologist, one prosthetics and orthotics specialist, and one CARF expert reviewer. This round resulted in two major changes. First, six items were added to address constructs not sufficiently covered, particularly ‘outcome.' To this end, we expanded and reclassified the generated items to cover the domains of Access (receipt of services), Structure (organization’s capability to provide services), Process (what happens during services), and Outcomes (results of services on the person served) (Table 1). Second, the Uncertain category of the original five-point Likert scale was removed, and a four-point scale was adopted to rate patient satisfaction (1 = Very dissatisfied, 2 = Dissatisfied, 3 = Satisfied, 4 = Very Satisfied), and an affirmative scale was used for the outcome domain and the global satisfaction items (1 = Strongly Disagree, 2 = Disagree, 3 = Agree, 4 = Strongly Agree). Additionally, there was minor rewording of some specific goal items for clarity.

Nine of the rehabilitation practitioners from the first round participated in the second round focus group, at which time a consensus was reached concerning the clarity, wording, and the importance of the 40 items that were used as the first version of the RH PSS. Topics addressed included access, structure, process, and outcome of care (ordered as follows: Pre-admission phase (5 items), Admission phase (12 items), Hospital environment (6 items), Discharge phase (5 items), Outcome (9 items), and Global items (3 items)). The remaining items were eight descriptive questions about patient demographics, previous rehabilitation experience, waiting time, discharge location, and one open-ended question that invited a free text response and patient comments.

### Step 3: Preliminary validation & survey finalization

This step, conducted at the end of 2010, included statistical analysis to establish the internal consistency for instrument reliability after 125 completed surveys were obtained, followed by detailed factor analysis, and reliability and validity testing using surveys completed by 182 participants. The survey (40 items) was subjected to the CTT procedures. The outcomes from the factor analyses showed that only 33 of the 40 survey items had a good correlation and a factor loading into five subscales in the range of 0.636–0.888. The subscales included: Access (7 items, α = 0.829), Structure (6 items, α = 0.815), Process (10 items, α = 0.914), Outcome (7 items, α = 0.806), Global (3 items, α = 0.852), and Entire Survey (33 items, α = 0.948). Further details of this stage are not mentioned for brevity because all measures were repeated but with a larger sample size (709 participants), which will be detailed in Step 4.

### Step 4: Survey implementation

#### Demographics of respondents

Survey participation by year was *N* = 182 (25.7%) in 2010, *N* = 172 (24.3%) in 2011, *N* = 159 (22.4%) in 2012, and *N* = 196 (27.6%) in 2013, with a total of *N* = 709 patients for the entire 4-year survey period. The ratio of males to females was 7:2; 51.7% of patients were under 30 years of age, and 83.2% of the participants were literate. Moreover, 281 (36.9%) completed the survey themselves, 146 (20.6%) were completed with assistance, and 282 (39.8%) were completed by proxy (Table 2).

**Table 2:** Demographics of patients completing the survey with valid percent (n = 709)

Demographic variables		No.	%
Session	2010	182	25.7
	2011	172	24.3
	2012	159	22.4
	2013	196	27.6
Age	6–12 years	44	6.2
	13–18 years	111	15.7
	19–30 years	213	30.0
	31–40 years	83	11.7
	41–50 years	66	9.3
	51–60 years	94	13.3
	>61	98	13.8
Gender	Male	546	77.0
	Female	163	23.0
Education	Illiterate	119	16.8
	Elementary	96	13.5
	Middle school	117	16.5
	High School	231	32.6
	College/ University	136	19.2
	Higher Education	10	1.4
Ward	Ward 1 (Mixed Male)	221	31.2
	Ward 2 (Mixed Female)	143	20.2
	Ward 3 (SCI Ward)	147	20.7
	Ward 4 (TBI Ward)	181	25.5
	Ward 6 (Pediatric Ward)	17	2.4
Person Completing Survey	Myself	281	39.6
	Myself - with help	146	20.6
	someone else on my behalf	282	39.8

#### Validity of the study tool

Principal Component Analysis extracted four factors with an eigenvalue of >1.0. The first factor explained a significant relationship with the 33 variables. The remaining three were ineffective. Further rotation with the Varimax method also explained four factors with 59.5% cumulative variance. The component loadings were acceptable and ranged from 0.477 to 0.836. The first component was named ‘Process’ and extracted 46.8% of the total variance and consolidated 11 variables characterized as Rehabilitation Care and Organization (what happens during services and includes items related to involvement, communication, courtesy and empathy, competency and confidence, promptness, routines and schedules). The second factor was named ‘Outcome’ and ‘Global’ domain, with 5.7% of total variance and consolidated 10 variables characterized (results of services on the person served) including six outcome variables and three global variables. The third factor was named ‘Structure’ and extracted 3.6% of the total variance and consolidated six variables characterized (organization’s capability to provide services). The fourth factor was ‘Access’ and extracted 3.4% of the total variance and consolidated six variables characterized (availability and receipt of services). One of the variables (Communication between the team) had a stronger loading on the first factor, ‘Outcome” (0.487), compared with ‘Process’ (0.443) but because of its conceptual relationship this variable was grouped with ‘Process’. Table 3 contains items defining each component along with their Varimax component loadings for the component on which the item loads most highly.

**Table 3:** Factor analysis: principal component analysis by Varimax Rotation Method for default >1 Eigenvalues

Component and item labels	I	II	III	IV
Factor 1: Process of Care				
1. Way team considered your needs	.65			
2. Courtesy and helpfulness of your team?	.65			
3. Info re: rights & responsibilities	.64			
4. Efforts made by your rehab team	.62			
5. Clarity of info about your admission	.61			
6. Knowing who to ask when you have questions	.60			
7. Explanation about your medications?	.59			
8. Way staff involved you in deciding about your program	.59			
9. Clarity of info about your rehab program	.58			
10. How soon did nursing respond to your call for help	.55			
11. The daily rehabilitation routine	.45			
12. Communication between the team	.44			
Factor 2: Outcome of Care				
1. I am confident in my ability to use the skills I was trained in		.66		
2. I was given adequate info re: changes to my home		.64		
3. I accomplished the goals set in my rehab		.57		
4. I / My family/caregiver received adequate information/ training in order to manage my condition at home.		.56		
5. I was given adequate info about medicines		.52		
6. My physical pain was controlled		.43		
Global Items				
7. I think this hospital has everything needed		.74		
8. I would recommend this program		.63		
9. Overall, I was satisfied with my experience		.59		
Factor 3: Structure of Care				
1. Privacy in your room			.84	
2. Peace and restfulness in your room			.80	
3. Cleanliness of the toilets and showers			.72	
4. Quality of food overall			.66	
5. Cleanliness of hospital			.56	
6. That your safety was not compromised			.49	
Factor 3: Access to Care				
1. Arrangement by the hospital for services/technical aids				.62
2. Way & time given to planning your return to home				.60
3. Waiting time to be admitted				.60
4. Meeting to discuss your discharge plans				.55
5. Arrangement for needed follow up plans				.51
6. Length of your rehab program				.45

#### Internal consistency and reliability of the RH PSS

Internal consistency for the RH PSS determined by Cronbach’s alpha exceeded 0.90 in each of the 33 independent parameters with the overall value for the model at 0.96. The correlation for each parameter was significant (*p* < 0.001). Therefore, we had sufficient evidence for all the items that exceeded to the minimum requisite number (10 × 33 = 330) deemed fit for the study. Cronbach’s alpha values for all the RH PSS domains ranged from 0.81 to 0.89 (Table 4).

**Table 4:** Internal consistency for the rehabilitation inpatient satisfaction survey (n = 709)

Sub-scale	Alpha	Item	corrected ‘r’	Alpha
Access (6 items)	0.815	Waiting time to be admitted	.432	.962
		Length of your rehab program	.705	.960
		Way & time given to planning your return to home	.663	.960
		Meeting to discuss your discharge plans	.689	.960
		Arrangement by the hospital for services/technical aids	.697	.960
		Arrangement for needed follow up plans	.725	.960
Structure (6 items)	0.842	Cleanliness of hospital	.595	.961
		Cleanliness of the toilets and showers	.537	.961
		Peace and restfulness in your room	.638	.960
		Privacy in your room	.634	.960
		Quality of food overall	.530	.962
		That your safety was not compromised	.655	.960
Process (12 items)	0.891	Info re: rights & responsibilities	.526	.961
		Clarity of info about your admission	.571	.961
		Way staff involved you in making decisions about your program	.677	.960
		Way team considered your needs	.715	.960
		Clarity of info about your rehab program	.690	.960
		Explanation about your medications	.700	.960
		Knowing who to ask when you have questions	.651	.960
		Courtesy and helpfulness of your team	.664	.960
		Efforts made by your rehab team	.667	.960
		How soon did nursing respond to your call for help	.677	.960
		Communication between the team	.670	.960
		The daily rehabilitation routine	.699	.960
Outcome (6 items)	0.872	My physical pain was controlled	.644	.960
		I was given adequate info about medicines	.697	.960
		I/My family/caregiver received adequate information/training in order to manage my condition and recover at home.	.718	.960
		I was given adequate info re: changes to my home	.694	.960
		I accomplished the goals set in my rehab	.643	.960
		I am confident in my ability to use the skills I was trained in	.632	.960
Global (3 items)	0.865	I think this hospital has everything needed	.722	.960
		I would recommend this program	.693	.960
		Overall, I was satisfied with my experience	.754	.960
**All Survey Items (33 items)**				**0.964**

#### Concurrent and predictive validity

Good predictive validity was demonstrated through the significant correlation between the global item “I would recommend this program to my family and friends” and overall satisfaction. The remaining 32 items of the RH PSS predicted 72% of the response variance in the global item (R2 = 0.786, adjusted R2 = 0.783). The table below presents the F-statistics of the multiple regression models for 33 items (Table 5). This was further confirmed using the Pearson’s correlation outlined in (Table 6).

**Table 5:** RH PSS: multiple regression-statistics for 33-item model

Model		Sum of Squares	Df	Mean Square	R Square	Adjusted R	F	Sig.
1	Regression	191.280	6	31.880	.786	.783	339.416	.000
	Residual	52.223	556	.094				
	Total	243.503	562					

a. Predictors: All other items (32) on the questionnaire

b. Dependent Variable: Global item “intent to recommend”

**Table 6:** RH PSS: bivariate correlation for the global item “intent to recommend” within domain and for 32-item model

Domain	Pearson’s correlation
Access domain	0.609^a^
Structure domain	0.303^b^
Process domain	0.553^a^
Outcome domain	0.461^a^
Global items	0.842^a^
All other items (whole survey)	0.652^a^

^a^Correlation is significant at the 0.01 level (2-tailed)

^b^Correlation is significant at the 0.05 level (2-tailed)

#### Correlation between inpatient demographics and satisfaction rate

There was no significant correlation between the RH PSS score and level of participants’ education, mode of survey completion or previous rehabilitation experience. Sex had a significant correlation with the global domain only (*p* = 0.044) (Table 7). There was a significant correlation between age and satisfaction, particularly in the Access (*p* = 0.010) and Process domains (*p* = 0.049); higher satisfaction rates were reported by older patients (>61) in these domains. Finally, a significant correlation was found between the ward of admission and satisfaction in four domains: Access (*p* = 0.003), Structure (*p* = 0.015), Process (*p* = 0.010), and Overall satisfaction rate (*p* = 0.016). Participants from Ward 1 consistently reported higher satisfaction, followed by Ward 3, then Ward 4; though Ward 3 reported the lowest satisfaction rate with the structure domain.

**Table 7:** Correlation between the study participants’ demographics and the satisfaction rate

Domain/Items	*P* values as determined by one way Anova					
	Age	Gender	Education	Ward	Previous Rehab Experience	Person completing the survey
Access	0.010	0.233	0.293	0.003	0.434	0.787
Structure	0.687	0.765	0.418	0.015	0.122	0.516
Process	0.049	0.122	0.281	0.010	0.120	0.760
Outcome	0.621	0.374	0.987	0.122	0.075	0.175
Global items	0.580	0.044	0.584	0.288	0.239	0.943
All other items (Entire survey)	0.210	0.560	0.685	0.016	0.168	0.521

## Discussion

The RH PSS is the first satisfaction survey designed for inpatient rehabilitation settings in Saudi Arabia with reported validity and reliability testing (Additional file 1: Appendix 1: The RH PSS Arabic Version; Additional file 2: Appendix 2: The RH PSS English Version). Our results demonstrated high internal consistency, validity, and reliability of the survey for use with patients admitted at KFMC-RH. The RH PSS-calculated Cronbach’s alpha indicates that RH PSS has a high degree of internal consistency and reliability. One important and distinctive feature of the RH PSS is that it assesses a wide array of items addressing four domains of rehabilitation care: Access, Structure, Process, and Outcomes, whereas traditional measures of patient satisfaction mainly address the Structure and Process of healthcare delivery [33]. This approach was adopted because of the literature support for such an approach that follows the classic Donabedian’s quality of care framework [6, 8, 27, 34]. Furthermore, the KFMC-RH opted to meet the CARF standards, which endorse such constructs.

The last question of the survey is an open-ended question inviting a free text response. It was found helpful in identifying ways to improve satisfaction, express feedback related to items that might not have be covered fully in the survey, and to overcome bias (e.g. social desirability or wanting to please service providers) in patient satisfaction surveys that has been reported by others [35].

Based on the results of preliminary testing and the recommendations of CARF consultants, the Uncertain category of the original five-level Likert scale was removed because this middle point provided respondents with moderate opinions a way to avoid answering. It has been noted that if the direction in which people are leaning on the issue is the type of information wanted, it is better not to suggest the middle ground and that in these cases no additional information is gained with a neutral middle point [6].

Another strength of the RH PSS, lending support to its content validity, lies in the process of survey development. The items were generated from information derived from different sources, including a literature review, in-depth interviews with patients, and multiple focus groups with rehabilitation professionals with diverse specialization.

Discipline-specific questions in the survey were present in the initial stages of survey development. However, because of the structure of the rehabilitation team, discipline-specific questions were viewed as problematic and subsequently rejected for two reasons. First, such questions would make the survey longer, which may be fatiguing and affect the response rate. For this reason, researchers have recommended that surveys should be brief (< 15 min.) [29, 36]. This is reflected in a study conducted by Heinemann and colleagues [15], who allocated 18 items of their 37-item questionnaire for care by specific discipline. Second, there is disagreement as to whether patients are able to differentiate between so many team members [3].

In a number of rehabilitation-related studies [16, 18, 21, 37], the interpersonal attributes of the therapists were attributed more to satisfaction than most functional outcomes. Nevertheless, others have reported that outcomes such as pain, active range of motion [17, 19, 20], and FIM discharge motor score predicted patient satisfaction [4]. In fact, Custer (2012) reported that functional status and gains in functional status, as assessed by FIM in combination with other independent variables, were the most robust and consistent predictors of satisfaction with rehabilitation in two subscales of clinical quality and client centeredness, each in slightly different ways [14]. For every one-point increase in FIM self-care scores (e.g., eating, bathing, toileting, dressing) at discharge, clients were 4.2% more likely to be satisfied on the client-centeredness subscale. Significant predictors of satisfaction with the clinical quality of rehabilitation included both the total FIM score with no self-care at discharge and the FIM self-care score at admission [14].

Despite the differing results, the literature shows a positive association with patient satisfaction and clinical effectiveness and therapy outcomes, and as such supports the inclusion of treatment outcomes as an important domain in a satisfaction survey. For the RH PSS, we opted to include treatment outcomes such as self-care items indirectly for three reasons. First, a large number of potential survey items might result in a longer survey, which could in turn affect the response rate [36]. Second, if degree of satisfaction is the person’s “pleasure or disappointment” based on their assessment of a service against their personal values and expectations [15] then the clarity of patient’s specific rehabilitation goals and outcomes would very much dictate their responses and impact the inclusion of specific outcome items in the survey. Al-Haidary et al. [38] questioned patient’s ability to form such clear goals and noted potential barriers to goal-setting such as lack of knowledge or understanding by the patient of his/her condition. This lack of understanding was partially attributed to the stigma attached to disability among some sectors of the Saudi population, who may see people with disability as helpless or dependent [38]. This is epitomized in the fact that walking/locomotion priority ranking corresponded well with its reported difficulty ratings. However, difficulty with self-care was not associated with its priority ranking, highlighting another cultural consideration that may be particular to the region, namely, the strong willingness among Saudi families to provide personal care or hire a caregiver to assist family members afflicted by injury or disease. Thus, it is possible that improvements in self-care ability may not be seen as a priority for some individuals with these cultural expectations, who may expect to receive help from others at home [38]. Third, considering the case mix and severity of the condition or functional limitation on admission, such direct questions might give a misperception that there is an expected level of improvement and thus influence the reported satisfaction for certain items negatively. One such example would be asking a patient who is unable to perform self-care tasks about their level of satisfaction with their ability to perform such task. Accordingly, the Outcome domain was covered with a question about pain, information about home medication and caregiver education, training to reduce the burden of care, home modification, the accomplishment of set program goals, and confidence in the ability to use the skills he or she was trained in.

The specific satisfaction rate with the KFMC-RH services obtained in the administration of this survey does not fall within the scope of this paper. However, descriptive statistics showed no significant statistical correlation between the RH PSS score and level of respondent's education, or previous rehabilitation experience. Sex had a correlation with the global domain only where females reported minimally higher satisfaction rate than males. However, sex had no significant correlation with other domains or the overall satisfaction rate. Older patients (>61) reported higher satisfaction rates in the Process and Access domains, which is similar to results reported in literature [4, 14].

### Study limitations

The initial validation stage involved a small sample of only 182 patients from one rehabilitation site in Saudi Arabia and so we caution generalizing these results to other patient populations or rehabilitation settings. Also, financial aspects are a common domain of patient satisfaction [13] yet were not addressed in the current survey development because KFMC-RH is a free-of-charge governmental rehabilitation hospital. Finally, the literature suggests a significant correlation between satisfaction level and therapy outcomes. However, this correlation is usually tested through a satisfaction measure and a functional outcome measure such as the FIM, administered separately. It would be interesting to capture and measure more of the outcome aspects directly via a satisfaction survey.

## Conclusions

The RH PSS is the first satisfaction survey designed for inpatient rehabilitation settings in Saudi Arabia to demonstrate reasonably reliability, and have good content, concurrent, and predictive validity. The authors recommend future research include larger sample sizes and multiple sites for nationwide validation.

## Additional files

Additional file 1: The RH PSS Arabic Version. Arabic version of the survey. (PDF 460 kb)

Additional file 2: The RH PSS English Version. English version of the survey. (PDF 540 kb)

## Abbreviations

CARF: Commission on Accreditation of Rehabilitation Facilities
CTT: Classical test theory
FIM: Functional Independence Measure
KFMC-RH: King Fahad Medical City Rehabilitation Hospital
RH PSS: King Fahad Medical City-Rehabilitation Hospital Patient Satisfaction Survey

## References

[1] Donabedian A (1966). Evaluating the quality of medical care. Milbank Mem Fund Q.

[2] Loblaw DA, Bezjak A, Bunston T (1999). Development and testing of a visit-specific patient satisfaction questionnaire: the Princess Margaret Hospital Satisfaction With Doctor Questionnaire. J Clin Oncol.

[3] Keith RA (1998). Patient satisfaction and rehabilitation services. YAPMR..

[4] Mancuso M, Smith P, Illig S, Granger CV, Gonzales VA, Linn RT (2003). Satisfaction with medical rehabilitation in patients with orthopedic impairment. YAPMR..

[5] Campbell J, Smith P, Nissen S, Bower P, Elliott M, Roland M (2009). The GP Patient Survey for use in primary care in the National Health Service in the UK--development and psychometric characteristics. BMC Fam Pract.

[6] uSPEQ Research Developent Team. uSPEQ Consumer Experience Survey Psychometric Evaluation. www.uspeq.org. Accessed 16 July 2016.

[7] Pascoe GC (1983). Patient satisfaction in primary health care: a literature review and analysis. Eval Program Plann.

[8] Hudak PL, Wright JG (2000). The characteristics of patient satisfaction measures. Spine.

[9] Sherman RO. Patient satisfaction now factors into Medicare reimbursement. Am Nurse Today. 2012;7

[10] Crow R, Gage H, Hampson S, Hart J, Kimber A, Storey L (2002). The measurement of satisfaction with healthcare: implications for practice from a systematic review of the literature. Health Technol Assess.

[11] Lewis JR (1994). Patient views on quality care in general practice: literature review. Soc Sci Med.

[12] Doering ER (1983). Factors influencing inpatient satisfaction with care. QRB Qual Rev Bull.

[13] Ware JE, Davies-Avery A, Stewart AL (1978). The measurement and meaning of patient satisfaction. Health Med Care Serv Rev.

[14] Custer MG. Developing a model of client satisfaction with a rehabilitation continuum of care [Internet]. ProQuest Dissertations Publishing; 2012. Available from: http://uknowledge.uky.edu/rehabsci_etds/7. Accessed 16 July 2016.

[15] Heinemann A, Bode R, Cichowski K, Kan E. Measuring patient satisfaction with medical rehabilitation. J Rehabil Outcome Meas. 1997;1:52–65.

[16] Stiller K, Cains G, Drury C. Evaluating inpatient satisfaction with a physiotherapy service: A rehabilitation centre survey. Int J Ther and Rehab. 2009;16(7):376–84.

[17] Bergés I-M, Ottenbacher KJ, Smith PM, Smith D, Ostir GV (2006). Perceived pain and satisfaction with medical rehabilitation after hospital discharge. Clin Rehabil.

[18] McKinnon AL (2009). Client Satisfaction with Physical Therapy Services. Phys Occup Ther Geriatr.

[19] Haase I, Lehnert-Batar A, Schupp W, Gerling J, Kladny B (2006). Factors contributing to patient satisfaction with medical rehabilitation in German hospitals. Int J Rehabil Res.

[20] Chiu TT, Lam T-H, Hedley AJ (2005). Correlation among physical impairments, pain, disability, and patient satisfaction in patients with chronic neck pain. YAPMR..

[21] Hush JM, Cameron K, Mackey M (2011). Patient satisfaction with musculoskeletal physical therapy care: a systematic review. Phys Ther.

[22] Ottenbacher KJ, Gonzales VA, Smith PM, Illig SB, Fiedler RC, Granger CV (2001). Satisfaction with medical rehabilitation in patients with cerebrovascular impairment. Am J Phys Med Rehabil.

[23] Sen S, Fawson P, Cherrington G, Douglas K, Friedman N, Maljanian R (2005). Patient satisfaction measurement in the disease management industry. Dis Manag.

[24] HCAHPS Survey [Internet]. Available from: http://www.hcahpsonline.org/files/2017_Survey%20Instruments_English_Mail.pdf. [cited 2009 Feb 18].

[25] Victorian Healthcare Experience Survey - VHES [Internet]. httpswww.health.vic.gov.auhospitals-and-health-servicesquality-safety-servicepatient-experience-survey. [cited 2009 Feb 18]. Available from: https://www2.health.vic.gov.au/hospitals-and-health-services/quality-safety-service/patient-experience-survey

[26] Patient Satisfaction Questionnaire from RAND Health [Internet]. rand.org. [cited 2009 Feb 18]. Available from: https://www.rand.org/health/surveys_tools/psq.html#citation

[27] Marshall GN, Hays RD. The Patient Satisfaction Questionnaire Short Form (PSQ-18) [Internet]. rand.org. Santa Monica; 1994 [cited 2009 Feb 18]. Available from: https://www.rand.org/pubs/papers/P7865.html

[28] Goldstein MS, Elliott SD, Guccione AA (2000). The development of an instrument to measure satisfaction with physical therapy. Phys Ther.

[29] Andresen EM (2000). Criteria for assessing the tools of disability outcomes research. YAPMR.

[30] Sitzia J (1999). How valid and reliable are patient satisfaction data? An analysis of 195 studies. Int J Qual Health Care.

[31] DePoy E, Gitlin LN. Introduction to Research: Understanding and Applying Multiple Strategies. 4 ed. St. Louis: Elsevier Mosby; 2010.

[32] DeVellis RF (2003). Scale Development**:** Theory and Applications.

[33] Paddock LE, Veloski J, Chatterton ML, Gevirtz FO, Nash DB (2000). Development and validation of a questionnaire to evaluate patient satisfaction with diabetes disease management. Diabetes Care.

[34] Nelson CW, Niederberger J (1990). Patient satisfaction surveys: an opportunity for total quality improvement. Hosp Health Serv Adm.

[35] Harding K, Taylor N (2010). Highly satisfied or eager to please? Assessing satisfaction among allied health outpatients. Int J Ther Rehabil.

[36] Dillman DA (2000). Mail and Internet Surveys.

[37] Beattie P, Turner C, Dowda M, Michener L, Nelson R (2005). The MedRisk Instrument for Measuring Patient Satisfaction With Physical Therapy Care: a psychometric analysis. J Orthop Sports Phys Ther.

[38] Al-Haidary H, Qannam H, Lam T (2015). Development of a rehabilitation goal menu for inpatients with neurological disorders: application in a Saudi Arabian context. Clin Rehabil.

