# Supplementary material for: Development of an Arabic inpatient satisfaction survey: application in acute medical rehabilitation setting in Saudi Arabia
Source: BMC Health Serv Res. 2017 Sep 18;17:664. doi: 10.1186/s12913-017-2596-2 (PMC5604416; doi:10.1186/s12913-017-2596-2)
Supplement: Supplementary file 1 — The RH PSS Arabic Version. Arabic version of the survey. (PDF 460 kb) [file 12913_2017_2596_MOESM1_ESM.pdf]

وزارة الصحة
مدينة الملك فهد الطبية
King Fahad Medical City
مستشفى التأهيل الطبي
Rehabilitation Hospital

استطلاع عن مدى رضا المريض
عن خدمات مستشفى التأهيل

إيمانا من إدارة مستشفى التأهيل لأهمية رأي المرضى الذين يتلقون العلاج من أجل تطوير الخدمات و تحسين جودة الرعاية المقدمة و الوصول إلى رضا المرضى لذا فإننا ندعوك عزيزي المريض للمشاركة في الإجابة عن الاستطلاع ويهدف هذا الاستطلاع لقياس مدى الرضا عن جودة الخدمات أثناء فترة إقامته بالمستشفى.

عزيزي المريض: هذا الاستطلاع سوف يسلم لك خلال آخر أسبوع من فترة إقامتك في المستشفى ونتوقع أن يستغرق الإجابة عن الأسئلة عشر دقائق تقريباً علماً بأنك غير ملزم على المشاركة إذ تعد مشاركتك عملاً تطوعيا تشكر عليه.

خصوصيتك محل احترامنا ونحن نحافظ عليها دائماً.
إن مشاركتك في هذا الاستطلاع أو عدم مشاركتك فيه سوف لن تؤثر بأي شكل من الأشكال على أي علاج قد تحتاجه في المستشفى مستقبلاً.

إذا كانت لديك أي أسئلة حول هذا الاستطلاع، أو إذا كنت لا ترغب في المشاركة:
نأمل التواصل مع الأخصائي الاجتماعي.

مع خالص شكرنا لحسن تعاونك،

إرشادات استطلاع الرأي

- يُعبأ هذا الاستبيان من قِبل المرضى المنومين بمستشفى التأهيل فقط.
- أجب عن جميع الأسئلة بوضع علامة √ على الإجابة المناسبة.
- يُرجى اختيار إجابة واحدة فقط في كل صف.

أسئلة عامة

أ.	العمر:	☐ 12-6	☐ 18-13	☐ 30-19	☐ 40-31	☐ 50-41	☐ 60-51	☐ أكثر من 61
ب.	الجنس:	☐ ذكر	☐ أنثى					
ت.	المستوى التعليمي:	☐ غير متعلم	☐ ابتدائي	☐ متوسط	☐ ثانوي	☐ جامعي	☐ تعليم عالي	
ث.	الجناح الذي أُدخلت به:	☐ جناح 1	☐ جناح 2	☐ جناح 3	☐ جناح 4	☐ جناح 5	☐ جناح 6	☐ جناح 7
ج.	التشخيص	☐ إصابات النخاع الشوكي	☐ سكتة/جلطة دماغية	☐ إصابة دماغ نتيجة حادث	☐ بتر الأطراف	☐ أخرى (حدد):		
ح.	الشخص الذي أكمل الاستبيان:	☐ أكملته بنفسي و بدون أية مساعدة	☐ أكملته بنفسي ولكن احدهم ساعدني في القراءة و كتابة أجوبتي	☐ شخصا آخر أكمل الاستبيان نيابة عن المريض				

تتعلق هذه الأسئلة عن مدى رضاك عن الخدمات التي قدمت لك بفترة ما قبل تنويمك في المستشفى					
	**ما مدى رضاك عن/حول:**	راضي تماماً	راضي	غير راضي	غير راضي إطلاقاً
1	المعلومات المقدمة حول حقوقك ومسئولياتك كمريض في المستشفى؟	☐	☐	☐	☐
2	وضوح الأهداف والتدخلات العلاجية والنتائج المتوقعة؟	☐	☐	☐	☐
3	الوقت الذي انتظرته حتى دخولك الى المستشفى؟	☐	☐	☐	☐

تتعلق هذه الأسئلة عن مدى رضاك عن الخدمات التي قدمت لك خلال تنويمك في المستشفى					
	**ما مدى رضاك عن/حول:**	راضي تماماً	راضي	غير راضي	غير راضي إطلاقاً
4	إشراك فريق التأهيل الطبي لك و/أو لافراد عائلتك في اتخاذ القرارات المتعلقة بوضع أهدافك العلاجية؟	☐	☐	☐	☐
5	أخذ فريقك التأهيلي لاحتياجاتك الشخصية بعين الاعتبار ؟	☐	☐	☐	☐
6	وضوح الشرح الذي تلقيته عن طبيعة برنامجك التأهيلي؟	☐	☐	☐	☐
7	وضوح أسباب إعطائك الدواء وأعراضه الجانبية؟	☐	☐	☐	☐
8	سهولة توفر من يجيبك عن تساؤلاتك حول علاجك؟	☐	☐	☐	☐
9	معاملة فريق التأهيل لك باحترام؟	☐	☐	☐	☐
10	جهد فريق التأهيل الطبي لتحقيق أهدافك العلاجية ؟	☐	☐	☐	☐
11	سرعة استجابة فريق التمريض لك عند حاجتك للمساعدة ؟	☐	☐	☐	☐
12	التواصل بين طاقم التأهيل فيما يخص علاجك مثل: زيارات الطاقم لك وعقدهم الاجتماعات معك و مع أسرتك...الخ	☐	☐	☐	☐
13	جدول تأهيلك اليومي والذي يشمل جداول العلاج ووقت الراحة والأنشطة الترفيهية...الخ.	☐	☐	☐	☐
14	مدة برنامجك التأهيلي خلال فترة تنويمك؟	☐	☐	☐	☐

تتعلق هذه الأسئلة بترتيبات الخروج من المستشفى					
	**ما مدى رضاك عن/حول:**	راضي تماماً	راضي	غير راضي	غير راضي إطلاقاً
15	الطريقة والزمن المعطى لترتيب خروجك من المستشفى.	☐	☐	☐	☐
16	اجتماعك مع فريق التأهيل لمناقشة خطة خروجك من المستشفى.	☐	☐	☐	☐
17	ترتيبات المستشفى لأي خدمات/ وسائل مساعدة تحتاجها تتعلق بمساعدتك للخروج.	☐	☐	☐	☐
18	ترتيبات المستشفى لأي برامج متابعة طبية تحتاجها بعد الخروج.	☐	☐	☐	☐

	تتعلق هذه الأسئلة في بيئة المستشفى وخدماته				
	**ما مدى رضاك عن/حول:**	راضي تماماً	راضي	غير راضي	غير راضي إطلاقاً
19	نظافة المستشفى بشكل عام ؟	☐	☐	☐	☐
20	نظافة دورات المياه ؟	☐	☐	☐	☐
21	الراحة والهدوء في الغرفة التي كنت تقيم فيها ؟	☐	☐	☐	☐
22	الخصوصية في الغرفة التي كنت تقيم فيها ؟	☐	☐	☐	☐
23	نوعية و جودة الطعام بصفة عامة ؟	☐	☐	☐	☐
24	الأخذ بوسائل السلامة في المستشفى ؟	☐	☐	☐	☐
	**تتعلق هذه الأسئلة بتقييمك العام للمستشفى و مدى استفادتك من برنامجك التأهيلي**				
	**خلال تواجدي في المستشفى:**	موافق بشدة	موافق	غير موافق	غير موافق إطلاقا
25	تلقيت علاجا فعالا للآلام التي كنت أشعر بها.	☐	☐	☐	☐
26	تلقيت شرحا كافيا عن الأدوية التي يجب أن أتناولها بعد خروجي.	☐	☐	☐	☐
27	تلقيت/ تلقت عائلتي تدريبا/ تعليما كافيا للتعامل مع حالتي وفترة شفائي في المنزل.	☐	☐	☐	☐
28	تلقيت شرحا كافيا فيما يخص المقترحات اللازمة لتعديل بيئة منزلي لمساعدتي بعد الخروج من المستشفى.	☐	☐	☐	☐
29	لقد حققت الاهداف التي تم وضعها لبرنامجي التأهيلي.	☐	☐	☐	☐
30	أنا قادر على القيام بالمهارات التي تعلمتها في المستشفى.	☐	☐	☐	☐
31	أعتقد بأن المستشفى فيها جميع ما يلزم لتقديم الخدمات التي أحتاجها.	☐	☐	☐	☐
32	سأوصي بهذا المستشفى لمن أعرفهم ممن يحتاجون إلى خدمات تأهيل.	☐	☐	☐	☐
33	بصورة عامة كانت خبرتي في هذا المستشفى مرضية.	☐	☐	☐	☐
-	**ما الذي يمكن للمستشفى القيام به لتحسين مستوى الرعاية والخدمات التي يقدمها وذلك من أجل تحقيقٍ أفضل الاحتياجات للمرضى؟**				

نشكرك على إكمالك هذا الاستبيان.

هذه المعلومات ستساعد المستشفى على تحسين خدماته المقدمة للمرضى.
