# Supplementary material for: Development of an Arabic inpatient satisfaction survey: application in acute medical rehabilitation setting in Saudi Arabia
Source: BMC Health Serv Res. 2017 Sep 18;17:664. doi: 10.1186/s12913-017-2596-2 (PMC5604416; doi:10.1186/s12913-017-2596-2)
Supplement: Supplementary file 2 — The RH PSS English Version. English version of the survey. (PDF 540 kb) [file 12913_2017_2596_MOESM2_ESM.pdf]

وزارة الصحة
مدينة الملك فهد الطبية
King Fahad Medical City
مستشفى التأهيل الطبي
Rehabilitation Hospital

REHABILITATION HOSPITAL
INPATIENT SATISFACTION SURVEY "RH PSS"

The "Inpatient Satisfaction Survey" is an initiative of the Rehabilitation Hospital originating from the hospital's strong belief in the importance of patients' feedback and participation in the hospitals' ongoing quality improvement endeavors and the hospital's determination to achieve high level of patients' satisfaction. As such, we cordially invite you to participate in this survey which aims to measure patients' satisfaction with the quality of their hospital experience.

You will receive this survey during your last week of your hospital stay. It is estimated to take about ten minutes to complete this survey. Your participation is important; however, we wish to indicate that your participation is entirely voluntary .

Your privacy will be protected at all times.
Your participation, or otherwise, in this survey will not influence any future hospital treatment you may require.

If you have any questions about the survey, or do not wish to participate, please contact the Hospital's Social Worker.

Thank you for your assistance and cooperation.

Questionnaire Instructions

- You should only fill this survey if you were admitted to the rehabilitation hospital only.
- Answer all the questions by checking the corresponding box.
- Please check/mark only one box on each row.

DESCRIPTIVE QUESTIONS

**I. Age:**	☐ 6-12	☐ 13-18	☐ 19-30	☐ 31-40	☐ 41-50	☐ 51-60	☐ 65+
**II. Gender:**	☐ Male	☐ Female					
**III. Education:**	☐ Illiterate	☐ Elementary school	☐ Middle School	☐ High school	☐ College/University	☐ Higher Education	
**IV. Which ward are you admitted to?**	☐ W 1	☐ W 2	☐ W 3	☐ W 4	☐ W 5	☐ W 6	☐ W 7

V. Person Completing Questionnaire

- ☐ Myself- person receiving service (no one helped)
- ☐ Myself- someone helped me read and write my answers
- ☐ Someone else on my behalf

These questions are about the time before your admission to the hospital.					
**How do you rate your satisfaction with:**		**Very satisfied**	**Satisfied**	**Dissatisfied**	**Very dissatisfied**
**1**	The information you were given about your rights and responsibilities as a patient?	☐	☐	☐	☐
**2**	The clarity of information you received about your admission, i.e., goals, interventions and expected outcomes?	☐	☐	☐	☐
**3**	The waiting time to be admitted?	☐	☐	☐	☐

These questions are about the time you were admitted in the hospital.					
**How do you rate your satisfaction with:**		**Very satisfied**	**Satisfied**	**Dissatisfied**	**Very dissatisfied**
**4**	The way staff involved you in making decisions about setting your treatment goals?	☐	☐	☐	☐
**5**	The way your rehab team considered your personal needs?	☐	☐	☐	☐
**6**	The clarity of information you received about your rehab program, i.e., treatment options, goals, and outcomes?	☐	☐	☐	☐
**7**	The explanation given to you about the purpose and side effects of your medications?	☐	☐	☐	☐
**8**	Knowing who to ask when you have questions about your treatment?	☐	☐	☐	☐
**9**	The courtesy and helpfulness of your rehabilitation team?	☐	☐	☐	☐
**10**	The efforts made by your rehab team to achieve your goals?	☐	☐	☐	☐
**11**	How soon did nursing respond to your call for help?	☐	☐	☐	☐
**12**	The communication between doctors, nurses and other hospital staff about your treatment, i.e., how well did your rehabilitation team work together with you to plan your care such as in ward rounds, one-one or family meetings…etc?	☐	☐	☐	☐
**13**	The daily rehabilitation routine –i.e., therapy schedules, rest time, recreational activities, etc.	☐	☐	☐	☐
**14**	The length of your rehabilitation program?	☐	☐	☐	☐

These questions are about preparing you for discharge from the hospital.					
**How do you rate your satisfaction with:**		**Very Satisfied**	**Satisfied**	**Dissatisfied**	**Very Dissatisfied**
**15**	The way and time given to planning your return to home?	☐	☐	☐	☐
**16**	The meeting your rehab team had with you to discuss your discharge plans?	☐	☐	☐	☐
**17**	The arrangement made by the hospital for any services/technical aids you needed in preparation for discharge?	☐	☐	☐	☐
**18**	The arrangement made by the hospital for needed follow up plan at the hospital as necessary?	☐	☐	☐	☐

These questions are about the hospitals' physical environment.				
**How do you rate your satisfaction with:**	**Very Satisfied**	**Satisfied**	**Dissatisfied**	**Very Dissatisfied**
**19** The cleanliness of the hospital?	☐	☐	☐	☐
**20** The cleanliness of the toilets and showers?	☐	☐	☐	☐
**21** The peace and restfulness in your room?	☐	☐	☐	☐
**22** The privacy in your room?	☐	☐	☐	☐
**23** The quality of food overall?	☐	☐	☐	☐
**24** That your safety was not compromised?	☐	☐	☐	☐

These questions are about the outcome of your program and your overall evaluation of hospital stay.				
**During this hospital stay:**	**Strongly Agree**	**Agree**	**Disagree**	**Strongly Disagree**
**25** My physical pain was controlled as well as possible.	☐	☐	☐	☐
**26** I was given adequate information about medicines I needed to take at home.	☐	☐	☐	☐
**27** I / My family/caregiver received adequate information/training to in order to manage my condition and recover at home.	☐	☐	☐	☐
**28** I was given adequate information about changes to my home that might be needed to help me.	☐	☐	☐	☐
**29** I accomplished the goals set in my rehabilitation program.	☐	☐	☐	☐
**30** I am confident in my ability to use the skills I was trained in.	☐	☐	☐	☐
**31** I think this hospital has everything needed to meet my needs.	☐	☐	☐	☐
**32** I would recommend this program to my family and friends.	☐	☐	☐	☐
**33** Overall, I was satisfied with my experience.	☐	☐	☐	☐

- What could the hospital do to improve the care and services it provides to better meet the needs of the patients?

Thank you for completing this questionnaire.

This feedback will help the hospital to improve its services for patients.
